# Palmitate Stimulates the Epithelial Sodium Channel by Elevating Intracellular Calcium, Reactive Oxygen Species, and Phosphoinositide 3-Kinase Activity

**DOI:** 10.1155/2018/7560610

**Published:** 2018-12-02

**Authors:** Qiu-Shi Wang, Chen Liang, Na Niu, Xu Yang, Xiao Chen, Bin-Lin Song, Chang-Jiang Yu, Ming-Ming Wu, Zhi-Ren Zhang, He-Ping Ma

**Affiliations:** ^1^Departments of Clinical Pharmacy and Cardiology, Harbin Medical University Cancer Hospital, Institute of Metabolic Disease, Heilongjiang Academy of Medical Science, Key Laboratories of Education Ministry for Myocardial Ischemia Mechanism and Treatment, Harbin 150000, China; ^2^Department of Physiology, Emory University School of Medicine, Atlanta, GA 30322, USA

## Abstract

Previous studies indicate that the epithelial sodium channel (ENaC) in the kidney is upregulated in diabetes mellitus. Here, we show that ENaC single-channel activity in distal nephron cells was significantly increased by palmitate, a free fatty acid which is elevated in diabetes mellitus. We also show that palmitate increased intracellular Ca^2+^ and that after chelating intracellular Ca^2+^ with BAPTA-AM, palmitate failed to affect ENaC activity. Treatment of the cells with 2-aminoethoxydiphenyl borate (2-APB, an inhibitor of IP_3_ receptors) abolished the elevation of both intracellular Ca^2+^ and ENaC activity. Treatment of the cells with apocynin (an NADPH oxidase inhibitor), dithiothreitol/NaHS (reducing agents), or LY294002 (a phosphoinositide 3-kinase (PI3K) inhibitor) prevented palmitate-induced ENaC activity, whereas thimerosal (an oxidizing agent) mimicked the effects of palmitate on ENaC activity. However, these treatments did not alter the levels of intracellular Ca^2+^, indicating that elevation of reactive oxygen species (ROS) and activation of PI3K are downstream of the signaling cascade. Since we have shown that ROS stimulate ENaC by activating PI3K, these data together suggest that palmitate first elevates intracellular Ca^2+^, then activates an NADPH oxidase to elevate intracellular ROS and PI3K activity, and finally increases ENaC activity via the activated PI3K.

## 1. Introduction

The epithelial sodium channel (ENaC), which is expressed primarily in the apical membrane of the epithelial cells lining the distal segment of nephrons, lung airways, alveoli, the descending colon, and endothelial cells [1], plays an important role in mediating Na^+^ entry into these cells. Na^+^ transport across the nephrons is critical for Na^+^ homeostasis and thus plays a vital role in maintaining salt balance and systemic blood pressure. Other more frequently observed pathological factors that alter ENaC activity may have much greater clinical significance treating hypertension. Hypertension is a common complication found in diabetes mellitus. Previous studies have shown that ENaC is activated in diabetic patients with nephropathy due to the elevation of ENaC-activating enzymes in the urine [2]. It has also been shown that high glucose stimulates ENaC expression in human cortical collecting duct cells [3]. These studies indicate that ENaC under diabetic conditions is not only activated by pathologically released enzymes in the urine but also activated either directly by hyperglycemia or indirectly by hyperglycemia-induced metabolic stress. In the patients with poorly controlled type 2 diabetes, plasma concentrations of free fatty acids (FFAs) are elevated [4]. FFAs are known to contribute to the pathogenesis of hypertension [5]. However, it remains unknown whether the elevated FFAs in diabetes mellitus cause hypertension by stimulating ENaC in the distal nephron cells.

FFAs generally refer to nonesterified fatty acids that are present in the blood. Palmitate, which is the main component of FFAs, can induce Ca^2+^ efflux from the endoplasmic reticulum (ER) [6, 7]. The redistribution of intracellular Ca^2+^, somehow, stimulates mitochondria to produce reactive oxygen species (ROS) [8, 9]. Several lines of evidence have shown that ROS not only stimulate phosphoinositide 3-kinase (PI3K) but also inactivate PTEN [10, 11]. Both can elevate phosphatidylinositol-3,4,5-trisphosphate (PIP_3_), which is a potent activator of ENaC [11–15]. These studies further suggest that FFAs, especially palmitate, may stimulate ENaC in the distal nephron to participate in the pathogenesis of hypertension in diabetes mellitus. Although ENaC can be specifically blocked by amiloride, amiloride may not be a useful drug to treat diabetes-induced hypertension because recent studies show that amiloride causes acute kidney injury [16]. Therefore, investigation of the signal transduction pathway for ENaC activation in diabetes mellitus becomes clinically significant. We have shown that the reducing agent, hydrogen sulfide, prevents ENaC activation by ROS [11, 17], which may provide an alternative approach to treat ROS-induced hypertension. In the present study, we show that palmitate stimulates ENaC by elevating intracellular Ca^2+^, ROS, and PI3K activity and that the stimulation can be corrected by hydrogen sulfide.

## 2. Materials and Methods

### 2.1. Cell Culture

A6 cells are an established renal cell line derived from distal nephron segments of *Xenopus laevis* and constitute an appropriate cell model for studying ENaC. A6 cells were purchased from the American Type Culture Collection (Rockville, MD, USA) and grown in medium consisting of 3 parts DMEM/F-12 (1 : 1) medium (Gibco, USA) and 1 part H_2_O, with 15 mM NaHCO_3_ (total Na^+^ = 101 mM), 2 mM L-glutamine, 10% fetal bovine serum (Invitrogen, USA), 25 units/ml penicillin, and 25 units/ml streptomycin, as previously described. A6 cells were cultured in plastic flasks in the presence of 1 *μ*M aldosterone at 26°C and 4% CO_2_. After the cells reached 70% confluence, they were subcultured on the polyester membranes of *Transwell* inserts (Corning Costar Co., USA) for confocal microscopy analysis or *Snapwell* inserts (Corning Costar Co, USA) for cell-attached patch-clamp experiments. To allow them to be fully polarized, cells were cultured for at least 2 to 3 weeks before performing the experiments [10].

### 2.2. Patch-Clamp Recording

ENaC single-channel currents were recorded using the cell-attached patch-clamp configuration with an Axopatch-200B amplifier (Axon Instruments, USA) as described previously [17]. A6 cells were thoroughly washed with a solution containing (in mM) 100 NaCl, 3.4 KCl, 1 CaCl_2_, 1 MgCl_2_, and 10 HEPES, adjusted to pH 7.4 with NaOH. This NaCl solution was used as the bath solution for recordings and to fill the electrodes. Reagents were added to the bath solution. The borosilicate glass electrodes had a tip resistance of 7–10 MΩ when filled with the NaCl solution. Experiments were conducted at room temperature (22–25°C). Data were acquired by applying a 0 mV pipette potential, sampled at 5 kHz and low-pass filtered at 1 kHz using Clampex 10.2 software (Molecular Devices, Sunnyvale, CA, USA). Prior to analysis, the single-channel traces were further filtered at 30 Hz. The total number of functional channels in the patch was determined by observing the number of peaks detected on the current amplitude histograms during a recording period of at least 10 min. The open probability (*P*_O_) values of ENaCs before and after chemical application were calculated using Clampfit 10.2 (Molecular Devices, Sunnyvale, CA, USA). Control ENaC activity was recorded for 2 min after the cell-attached mode was established and ENaC activity stabilized. A single patch was typically recorded for at least 30 min before any experimental manipulation.

### 2.3. Confocal Laser Scanning Microscopy Analysis

Confocal microscopy (Olympus Fluoview 1000, Japan) studies were performed as previously described [11, 17]. A6 cells were washed twice with the same NaCl solution described above prior to the performance of any experiments. Immediately following experimental manipulation, the polyester membrane support was quickly excised and mounted on a glass slide with a drop of NaCl solution to keep the cells alive. A6 cells grown on Transwell inserts were loaded with 2.5 *μ*M 5-(and-6)-carboxy-2′,7′-dichlorodihydrofluorescein diacetate (carboxy-H_2_DCFDA), a membrane-permeable ROS-sensitive fluorescent probe (Invitrogen, USA) that becomes fluorescent when oxidized. Prior to the application of palmitate, A6 cells were treated with an iron chelator, 50 *μ*M 2,2′-dipyridyl, for 3 min [17]. Labeled cells were washed twice in modified DPBS prior to confocal microscopy analysis. ROS levels were measured based on fluorescence intensity. To determine intracellular Ca^2+^ levels, A cells were incubated with 5 *μ*M Fluo-3, AM, a fluorescent Ca^2+^ indicator, for 60 min [18]. Confocal microscopy XY scanning of the cells was accomplished within 5–15 min. In each set of experiments, images were taken using the same parameter settings.

### 2.4. Chemicals and Reagents

Unless otherwise noted, all chemicals and reagents were purchased from Sigma-Aldrich (St. Louis, MO, USA). All solutions were premade and stored in a −20°C freezer or made fresh before use. Palmitic acid was purchased from Sigma, and BSA (FFA-free) was purchased from Roche. Palmitic acid was dissolved in 0.1 M NaOH at 70°C and then complexed with 10% BSA at 55°C for 10 min to achieve a final palmitate concentration of 0.3 mM. Stock solutions of 3 mM palmitate with 10% BSA and of a 10% BSA control were prepared 1 day before the experiments.

### 2.5. Data Analysis

Data are presented as the mean ± S.E. Statistical analysis was performed using SigmaPlot and SigmaStat Software (Jandel Scientific, CA, USA). Student's *t*-tests were used to compare pre- and posttreatment activities. Analysis of variance (ANOVA) was used to perform multiple comparisons among various treatment groups. Differences were considered statistically significant at *P* < 0.05.

## 3. Results

### 3.1. Palmitate Increases ENaC Activity and Elevates Intracellular Ca^2+^

**Figure 1:**
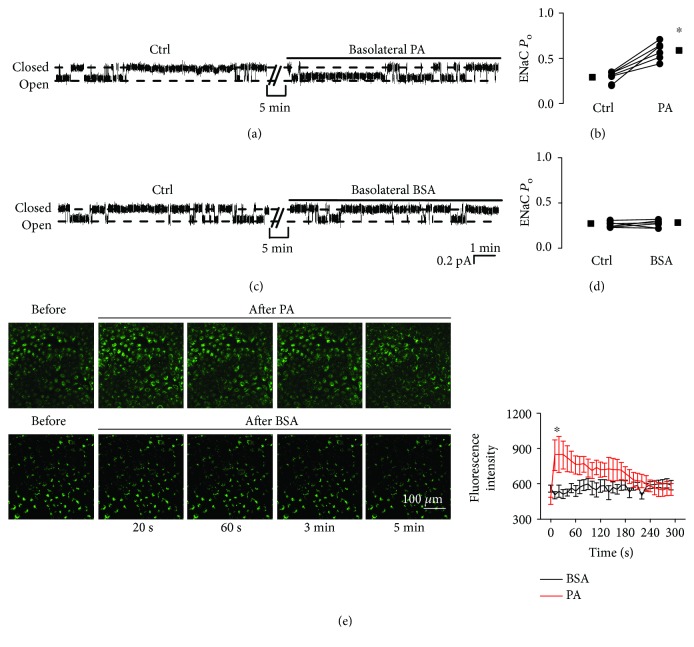
Palmitate increases ENaC activity and elevates intracellular Ca^2+^ levels in A6 cells. (a and c) Representative ENaC single-channel current recorded from A6 cells before and after application of either 0.3 mM palmitate (PA) or 2% BSA to the basolateral bath. (b and d) Summary plots show that palmitate significantly increased ENaC *P*_O_ (*n* = 7 for each experimental treatment; ^∗^*P* < 0.05), but BSA did not affect ENaC *P*_O_. (e) Left panel shows representative confocal microscopy images of A6 cells, which were loaded with Fluo-3, AM (a Ca^2+^ indicator), before and after application of either palmitate or BSA to the basolateral bath. Right panel shows summary plots of fluorescence intensity of Fluo-3 indicating the levels of intracellular Ca^2+^ (*n* = 7 for each individual experimental treatment; ^∗^*P* < 0.05). Note: in patch-clamp experiments, all the responses had approximately 5 min latency. Therefore, in all the figures, we omitted 5 min recordings after each experimental manipulation.

**Figure 2:**
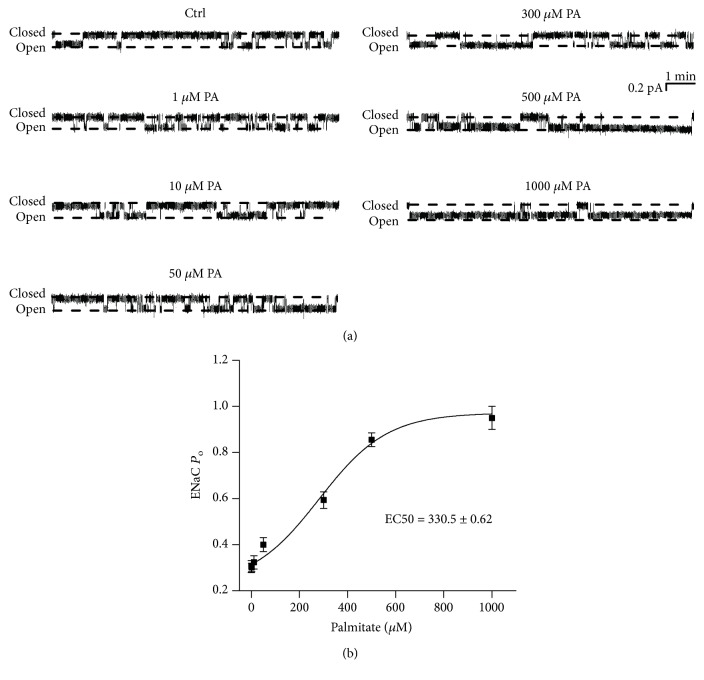
Palmitate (PA) stimulates ENaC activity in a dose-dependent manner. (a) Representative ENaC single-channel currents recorded in A6 cells under control conditions or treated with palmitate at concentrations of 1, 10, 50, 300, 500, and 1000 *μ*M. (b) ENaC *P*_O_ was plotted as a function of each corresponding concentration of palmitate and fitted with pharmacology standard curves. Analysis was performed using SigmaPlot (*n* = 6 for each data point).

To investigate whether palmitate alters ENaC activity, we performed cell-attached patch-clamp experiments. Because plasma palmitate levels are elevated in diabetic patients [4], palmitate was applied to the basolateral bath to mimic the *in vivo* mode of plasma palmitate delivery. Single-channel ENaC currents were recorded for at least 30 min in each experiment. Addition of palmitate (0.3 mM) to the basolateral bath significantly increased ENaC *P*_O_ from 0.29 ± 0.01 (control before the addition) to 0.59 ± 0.04 (after addition of palmitate) (*P* < 0.05; *n* = 7; Figures 1(a) and 1(b)). Since palmitate was dissolved in a solution containing 2% of BSA, as a control, we also applied 2% BSA to the basolateral bath; it did not affect ENaC *P*_O_ (0.27 ± 0.01 versus 0.28 ± 0.01; *P* > 0.05; *n* = 7; Figures 1(c) and 1(d)). Recent studies have shown that elevation of intracellular Ca^2+^ near the basolateral membrane stimulates ENaC [19]. Therefore, next, we tested if palmitate can increase intracellular Ca^2+^ in A6 cells. Application of palmitate (0.3 mM) significantly increased intracellular Ca^2+^ within approximately 20 s, followed by a gradual decline, but the increase remained significantly higher than the levels before the treatment for at least 3 min. In contrast, the exposure of cells to 2% BSA did not alter intracellular calcium levels (Figure 1(e)). ENaC activity was gradually elevated by increasing the doses of palmitate, from 0.28 ± 0.03 (control) to 0.95 ± 0.05 (1000 *μ*M) (Figure 2(a)). The calculated mean *P*_O_ of ENaC was plotted as a function of different doses of palmitate and fitted with dose-response curve analysis (the solid black line), having an EC50 of 330.5 ± 0.62 *μ*M for palmitate to activate ENaC (Figure 2(b); *n* = 6 for each data point). Therefore, 300 *μ*M of palmitate (a dose close to EC50) was used for the rest of the experiments.

### 3.2. Palmitate Stimulates ENaC via a Ca^2+^-Dependent Pathway

**Figure 3:**
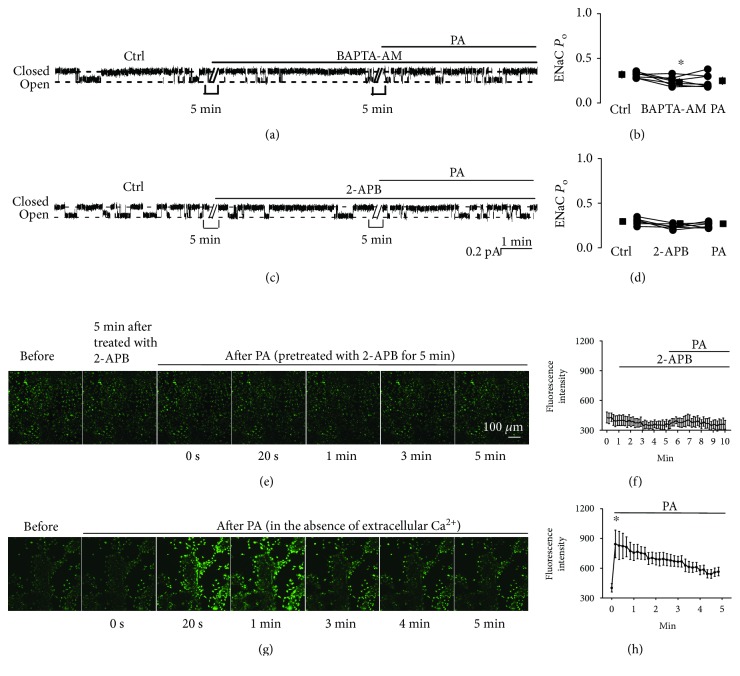
Palmitate stimulates ENaC via a Ca^2+^-dependent mechanism. (a) Representative ENaC single-channel current recorded from an A6 cell before and after application of BAPTA-AM (a membrane-permeable Ca^2+^ chelator; 10 *μ*M) and palmitate (0.3 mM) to the basolateral bath. (b) Summarized ENaC *P*_O_ before and after the application of different reagents (*n* = 6; ^∗^*P* < 0.05, compared with the control). (c) Representative single-channel ENaC current recorded from an A6 cell before and after application of 2-APB (an inhibitor of IP3 receptors which inhibits store-operated calcium release; 100 *μ*M) and palmitate (0.3 mM) to the basolateral bath. (d) Summarized ENaC *P*_O_ before and after application of different reagents (*n* = 6; *P* > 0.05, compared with the control). (e) Representative confocal microscopy images of A6 cells, which were loaded with Fluo-3, AM (a Ca^2+^ indicator), under control conditions (before), 5 min after treatment with 2-APB, and after application of palmitate to the basolateral bath. (f) Summary plots of fluorescence intensity of Fluo-3 indicating the levels of intracellular Ca^2+^. Each point was averaged from 8 images. Data are from six separate experiments. (g) Representative confocal microscopy images of A6 cells, which were loaded with Fluo-3, AM, before (in the absence of extracellular Ca^2+^) and after application of palmitate to the basolateral bath. (h) Summary plots of fluorescence intensity of Fluo-3 indicating the levels of intracellular Ca^2+^. Each point was averaged from 8 images. Data are from six separate experiments.

To test the effect of intracellular Ca^2+^ on palmitate-induced ENaC activity, we treated A6 cells with BAPTA-AM (a membrane-permeable Ca^2+^ chelator) for 5 min before the addition of palmitate. Basolateral addition of palmitate no longer increased ENaC activity in the presence of BAPTA-AM, albeit BAPTA-AM slightly but significantly decrease ENaC activity. ENaC *P*_O_ was 0.32 ± 0.02 (control), 0.25 ± 0.03 (BAPTA-AM), and 0.23 ± 0.02 (palmitate) (*P* < 0.05; *n* = 6; Figures 3(a) and 3(b)). To determine whether palmitate induces Ca^2+^ release from the endoplasmic reticulum (ER), we treated A6 cells with 2-APB, an inhibitor of IP3 receptors, for 5 min before the addition of palmitate. The data show that palmitate no longer alters ENaC activity in the presence of 2-APB. ENaC *P*_O_ was 0.27 ± 0.02 (control), 0.30 ± 0.02 (2-APB), and 0.27 ± 0.02 (palmitate) (*P* > 0.05; *n* = 6; Figures 3(c) and 3(d)). Similarly, palmitate also no longer increases intracellular Ca^2+^ after treatment of the cells (Figures 3(e) and 3(f)). To further determine whether palmitate induces Ca^2+^ release from ER, we examined the effect of palmitate on the levels of intracellular Ca^2+^ in the absence of extracellular Ca^2+^. The results show that in the absence of extracellular Ca^2+^, palmitate still elevated intracellular Ca^2+^ (Figure 3(g) and 3(h)). These results suggest that palmitate stimulates ENaC probably via a pathway associated with Ca^2+^ release from ER.

### 3.3. NaHS Reverses Palmitate-Induced Oxidative Stress and ENaC Activation

**Figure 4:**
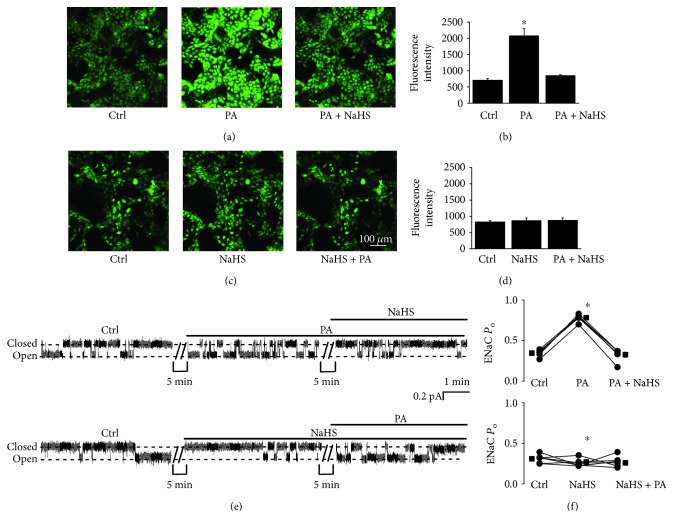
NaHS abolishes palmitate-induced oxidative stress and ENaC activation. (a and c) Confocal microscopy images of A6 cells loaded with DCF, an ROS indicator, before and either after application of palmitate (0.3 mM) first, then NaHS (0.1 mM), or after application of NaHS (0.1 mM) first, then palmitate (0.3 mM), to the cells. (b and d) Summary plots of fluorescent intensity under each condition as indicated. Data are from six independent paired experiments (*n* = 6 for each individual experimental set; ^∗^*P* < 0.05, compared with the control group). (e) Representative ENaC single-channel current recorded from two A6 cells before and either after addition of 0.3 mM palmitate first, then 0.1 mM NaHS (upper trace), or after addition of 0.1 mM NaHS first, then 0.3 mM palmitate, to the basolateral bath. (f) Summarized ENaC *P*_O_ under each condition, as indicated (*n* = 6 for each individual experimental treatment; ^∗^*P* < 0.05, compared with the control treatment group).

**Figure 5:**
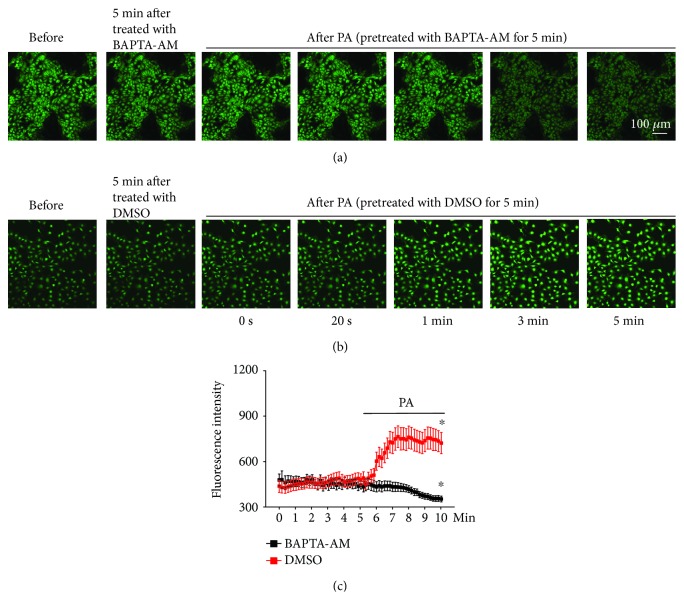
Palmitate elevates intracellular ROS via a Ca^2+^-dependent mechanism. (a) Representative confocal microscopy images of A6 cells, which were loaded with DCF (an ROS indicator), under control conditions (before), 5 min after treatment with BAPTA-AM (10 *μ*M), and after application of palmitate to the basolateral bath in the presence of BAPTA-AM. (b) A6 cells under control conditions (before), 5 min after treatment with DMSO (vehicle control), and after application of palmitate to the basolateral bath. (c) Summary plots of fluorescence intensity of DCF indicating the levels of intracellular ROS (*n* = 6; ^∗^*P* < 0.05, compared with the control). Each point was 8 cells randomly selected from each image in six sets of separate experiments.

To determine if palmitate can induce oxidative stress, intracellular ROS were measured. The data show that palmitate did elevate intracellular ROS and that the elevation was abolished by NaHS, no matter whether palmitate or NaHS was first added to the basolateral bath (Figures 4(a)–4(d)). In parallel, palmitate-induced ENaC activity was also abolished by NaHS (Figures 4(e) and 4(f)). As shown in Figures 4(e) and 4(f), we repeatedly found that addition of 0.3 mM palmitate to the basolateral bath significantly increased ENaC *P*_O_ from 0.35 ± 0.02 to 0.78 ± 0.02 (*P* < 0.05; *n* = 6). In the presence of palmitate, application of 0.1 mM NaHS to the basolateral bath reversed the effect. ENaC *P*_O_ was reduced, from 0.78 ± 0.02 to 0.33 ± 0.03 (*P* < 0.05; *n* = 6). Conversely, in the presence of 0.1 mM NaHS, palmitate failed to increase ENaC activity. ENaC *P*_O_ remained unchanged, 0.25 ± 0.03 (NaHS) vs. 0.23 ± 0.03 (NaHS plus palmitate) (*P* > 0.05; *n* = 6). However, NaHS only slightly decreased ENaC *P*o, 0.31 ± 0.02 (control) vs. 0.25 ± 0.03 (NaHS) (*P* < 0.05; *n* = 6). These results suggest that H_2_S exerts a strong protective effect against the palmitate-induced ENaC activity in A6 cells by reducing oxidative stress induced by palmitate. To determine if elevation of intracellular Ca^2+^ occurs before the oxidative stress, we pretreated the cells with BAPTA-AM and then applied palmitate to the cells. The data show that BAPTA-AM abolished palmitate-induced elevation of intracellular ROS (*P* < 0.05; *n* = 6; Figures 5(a)–5(c)). These results suggest that ROS, as a downstream signaling molecule of intracellular Ca^2+^, mediate the stimulation of ENaC by palmitate.

### 3.4. Apocynin Attenuates Palmitate-Induced ENaC Activity but Does Not Affect Intracellular Ca^2+^

**Figure 6:**
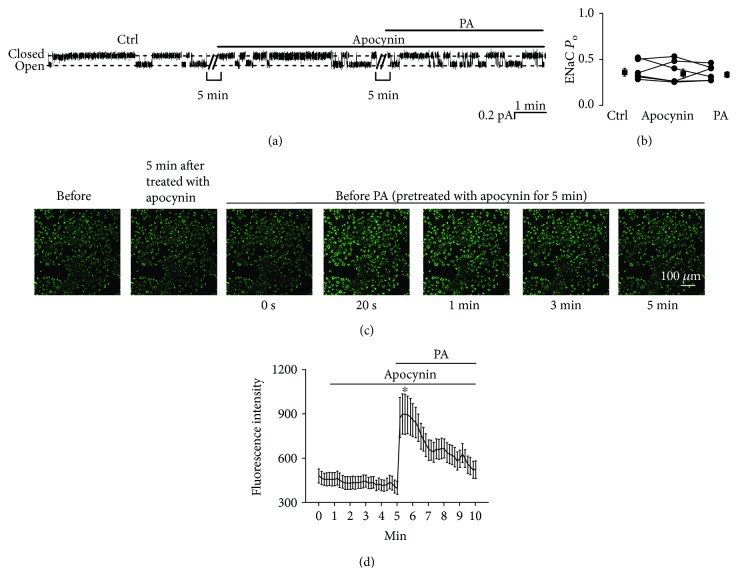
Apocynin abolishes palmitate-induced ENaC activity but does not affect intracellular Ca^2+^. (a) Representative ENaC single-channel current recorded from an A6 cell before and after addition of 0.1 mM apocynin first, then 0.3 mM palmitate to the basolateral bath. (b) Summarized ENaC *P*_O_ before and after application of different reagents (*n* = 6; *P* > 0.05). (c) Representative confocal microscopy images of A6 cells, which were loaded with Fluo-3, AM (a Ca^2+^ indicator), under control conditions (before), 5 min after treatment with apocynin, and after application of palmitate to the basolateral bath. (d) Summary plots of fluorescence intensity of Fluo-3 indicating the levels of intracellular Ca^2+^. (*n* = 6; ^∗^*P* < 0.05, compared with the control).

To determine whether palmitate stimulates ENaCs by activating an NADPH oxidase, we pretreated A6 cells with 0.1 mM apocynin, an NADPH oxidase inhibitor, for 5 min. As shown in Figure 6(a), after pretreatment, palmitate no longer affects ENaC activity. ENaC *P*_O_ was 0.36 ± 0.04 (control), 0.34 ± 0.05 (apocynin), and 0.33 ± 0.03 (apocynin plus palmitate) (*P* > 0.05; *n* = 6; Figure 6(b)). However, apocynin did not alter the elevation of intracellular Ca^2+^ induced by palmitate (Figures 6(c) and 6(d)). These data suggest that palmitate stimulates ENaC via an NADPH oxidase-mediated production of ROS through elevation of intracellular Ca^2+^.

### 3.5. Palmitate Stimulates ENaC via a Redox-Dependent Mechanism

**Figure 7:**
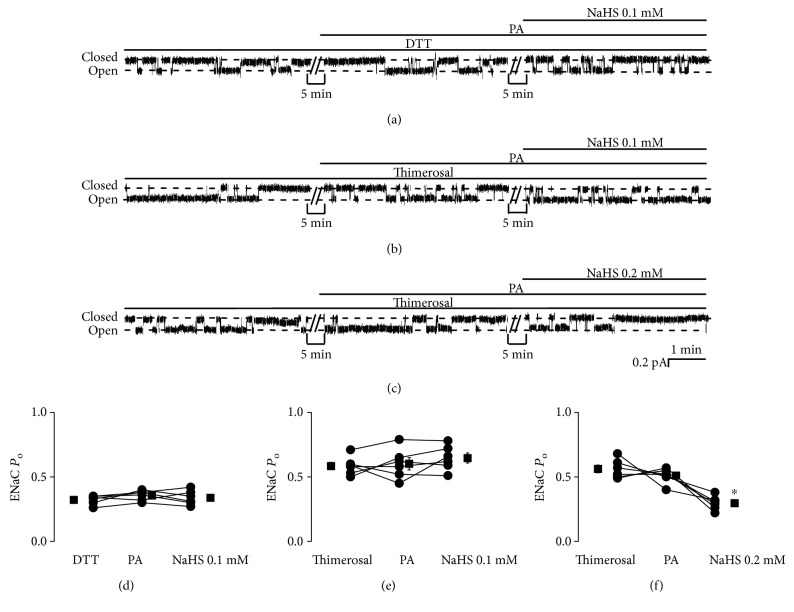
Palmitate stimulates ENaC via a redox-dependent mechanism. (a–c) Representative ENaC single-channel current recorded from A6 cells. Either DTT (a reducing agent, 1 mM) or thimerosal (an oxidizing agent, 100 *μ*M) was first added to the basolateral bath. Then, palmitate was added before application of NaHS (0.1 mM) to the basolateral bath. (d–f) Summarized ENaC *P*_O_ under each condition, as indicated (*n* = 6 for each individual experimental set; ^∗^*P* < 0.05 compared with thimerosal and palmitate treatments).

To determine if palmitate stimulates ENaC via a redox-dependent mechanism, either DTT, a reducing agent, or thimerosal, an oxidizing agent, was used to pretreat the cells before addition of palmitate. After pretreatment with DTT (1 mM), palmitate no longer affects ENaC activity. Addition of NaHS (0.1 mM) also did not affect ENaC activity. ENaC *P*_O_ was 0.32 ± 0.01 (DTT), 0.36 ± 0.02 (DTT plus palmitate), and 0.34 ± 0.02 (DTT plus palmitate plus NaHS) (*P* > 0.05; *n* = 6; Figures 7(a) and 7(d)). After treatment with thimerosal (100 *μ*M), ENaC activity was increased, but addition of palmitate did not cause any additive effects, and 0.1 mM NaHS did not reverse the effects of thimerosal. ENaC *P*_O_ was 0.58 ± 0.03 (thimerosal; compared to control as shown above, *P* < 0.05), 0.60 ± 0.05 (thimerosal plus palmitate), and 0.65 ± 0.04 (thimerosal plus palmitate plus NaHS) (*P* > 0.05; *n* = 6; Figures 7(b) and 7(e)). However, with the same treatment of thimerosal and palmitate, a higher concentration of NaHS (0.2 mM) did reduce ENaC activity. ENaC *P*_O_ was 0.56 ± 0.03 (thimerosal), 0.51 ± 0.02 (thimerosal plus palmitate), and 0.30 ± 0.02 (thimerosal plus palmitate plus NaHS) (*P* < 0.05; *n* = 6; Figures 7(c) and 7(f)). These results suggest that palmitate stimulates ENaC via a redox-dependent mechanism.

### 3.6. LY294002 Attenuates Palmitate-Induced ENaC Activity but Does Not Affect Intracellular Ca^2+^

**Figure 8:**
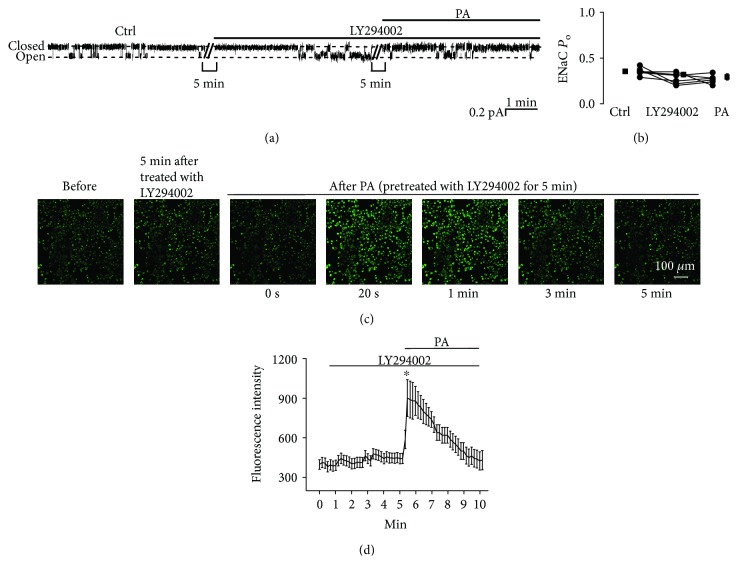
LY294002 abolishes palmitate-induced ENaC activity but does not affect intracellular Ca^2+^. (a) Representative ENaC single-channel current recorded from an A6 cell before and after addition of 5 *μ*M LY294002 first and then 0.3 mM palmitate to the basolateral bath. (b) Summarized ENaC *P*_O_ under each condition, as indicated (*n* = 6; *P* > 0.05). (c) Representative confocal microscopy images of A6 cells, which were loaded with Fluo-3, AM (a Ca^2+^ indicator), under control conditions (before), 5 min after treatment with LY294002, and after application of palmitate to the basolateral bath. (d) Summary plots of fluorescence intensity of Fluo-3 indicating the levels of intracellular Ca^2+^. Each point was averaged from 8 samples. Data are from 6 independent experiments (*n* = 6; ^∗^*P* < 0.05, compared with the control).

Our previous data have shown that ROS stimulate ENaC by increasing apical PI(3,4,5)P_3_ via activation of PI3K [10, 17]. To determine whether PI3K mediates the effects of palmitate on ENaC activity, A6 cells were pretreated with 5 *μ*M LY294002, a PI3K inhibitor, before addition of 0.3 mM palmitate to the basolateral bath. As shown in Figures 8(a) and 8(b), ENaC *P*_O_ was slightly decreased from 0.35 ± 0.02 to 0.32 ± 0.03 by LY294002, whereas palmitate failed to increase ENaC activity in cells pretreated with LY294002. ENaC *P*_O_ remained unchanged, 0.32 ± 0.03 (LY294002) vs. 0.29 ± 0.03 (LY294002 plus palmitate; *P* > 0.05; *n* = 6). However, LY294002 did not affect palmitate-induced elevation of intracellular Ca^2+^ (*P* < 0.05; *n* = 6; Figures 8(c) and 8(d)). These data suggest that the stimulatory effect of basolateral palmitate on ENaC is dependent on Ca^2+^-dependent activation of PI3K.

## 4. Discussion

Our major findings in this study are as follows: (1) palmitate stimulates ENaC by elevating intracellular Ca^2+^ and ROS, (2) NaHS reverses the effects of palmitate on ENaC activity by reducing the palmitate-induced accumulation of intracellular ROS, (3) the inhibitory effect of NaHS on palmitate-induced ENaC activity is exerted through its reducing action, and (4) palmitate stimulates ENaC by increasing PI3K activity.

Previous studies have shown that palmitate is able to induce *β*-cell apoptosis [20]. However, our data show that palmitate did not induce any type of cell death, even though the cells were incubated with 0.3 mM palmitate for up to 24 h (data not shown). Therefore, the effects of palmitate on ENaC were likely not due to nonspecific effects on cell viability. Previous studies have shown that palmitate in the presence of Ca^2+^ can form pores in the membrane for Ca^2+^ influx [21]. It is possible that a high concentration is required for palmitate to pass through the Transwell membrane, to be incorporated into the basolateral membrane of A6 cells, and to finally form Ca^2+^-permeable pores. However, we do not favor this possibility because our data show that the increases in intracellular Ca^2+^ can be abolished by 2-APB, an inhibitor of the IP_3_ receptor which is located in the ER membrane. Therefore, we argue that a high concentration may be required for palmitate to pass through the basolateral membrane of A6 cells to finally target the ER membrane to cause Ca^2+^ release from ER. Previous data suggest that elevation of intracellular Ca^2+^ inhibits ENaC by activating protein kinase C [22]. However, recent data indicate that elevation of intracellular Ca^2+^ near the apical membrane inhibits ENaC through purinergic signaling via the P2Y2 receptor on the apical membrane [23]. In contrast, basolateral elevation of intracellular Ca^2+^ near the basolateral membrane stimulates ENaC via mitochondria sequester intracellular Ca^2+^, creating intracellular Ca^2+^ signaling microdomains [19].

Here, we show that the released Ca^2+^ causes an NADPH oxidase-dependent elevation of ROS. This is not surprising because previous studies have already shown that there is an interaction between ER Ca^2+^ and mitochondrial ROS in pulmonary arterial smooth muscle cells [24]. Since Ca^2+^ also stimulates NADPH oxidase 4 (NOX-4) in the mitochondria [25], we have previously shown that excess mitochondrial ROS significantly increased ENaC activity [19]. We argue NOX-4 may also contribute to the elevated mitochondrial ROS induced by palmitate. It would be interesting to examine whether the mitochondrial NOX-4 mediates palmitate-induced ENaC activity in A6 cells. Our previous studies have shown that hydrogen peroxide, an ROS, does not alter ENaC activity in excised inside-out patches [10]. Therefore, it is unlikely that palmitate-induced elevation of intracellular ROS stimulates ENaC by directly oxidizing ENaC. Here, we show that inhibition of PI3K can abolish the activation of ENaC by palmitate, since it is well known that PIP_3_, a product of PI3K, is a strong activator of ENaC [11–15]. We favor the notion that palmitate stimulates ENaC via a pathway associated with Ca^2+^-initiated elevation of intracellular ROS and the downstream activation of PI3K because LY294002 did not affect palmitate-induced elevation of intracellular Ca^2+^. Although there is no direct evidence to show Ca^2+^ stimulate PI3K, our data have shown that palmitate mediates elevation of intracellular Ca^2+^ and subsequently causes ROS elevation. Other's data shows that ROS increased the levels of PI3K activity [26]. Previous studies show the *β* and *γ* ENaC subunits are modified by Cys palmitoylation through fatty acid-exchange chemistry experiment and *β*-subunit palmitoylation is associated with an increase in channel activity [27]. Our data further demonstrated besides directly modulating the channel gating, palmitate could also increase ENaC activity by regulating the intracellular signaling process.

**Figure 9:**
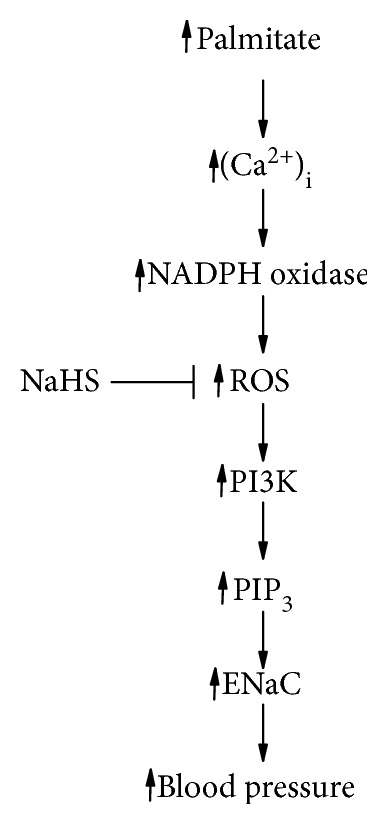
The proposed mechanism by which PA upregulates ENaC probably via a sequential pathway associated with elevation of intracellular Ca^2+^, ROS via an NADPH oxidase, and PIP3 via PI3K to elevate blood pressure.

Overall, we proposed the underlying mechanism by which PA upregulates ENaC probably via a sequential pathway associated with elevation of intracellular Ca^2+^, ROS via an NADPH oxidase, and PIP3 via PI3K to elevate blood pressure (Figure 9). The important finding of this study is that NaHS can abolish the activation of ENaC by palmitate, a major FFA which is elevated in diabetes [4]. However, whether NaHS can be used to treat diabetes-induced hypertension remains to be studied.

## 5. Conclusion

Palmitate stimulates ENaC activity in A6 cells via Ca^2+^-dependent activation of NADPH oxidase, production of ROS, and activation of PI3K. The palmitate-induced stimulation of ENaC can be reversed by NaHS.

## Data Availability

The data used to support the findings of this study are available from the corresponding author upon request.

## Abbreviations

2-APB: 2-Aminoethoxydiphenyl borate
ENaC: Epithelial sodium channel
FFAs: Free fatty acids
PA: Palmitate
PI3K: Phosphoinositide 3-kinase
PIP_3_: Phosphatidylinositol-3,4,5-trisphosphate
*P*_O_: Open probability
ROS: Reactive oxygen species.
